# Biallelic Variants in *EPHA2* Identified in Three Large Inbred Families with Early-Onset Cataract

**DOI:** 10.3390/ijms221910655

**Published:** 2021-09-30

**Authors:** Priya Jarwar, Shakeel Ahmed Sheikh, Yar Muhammad Waryah, Ikram Uddin Ujjan, Saima Riazuddin, Ali Muhammad Waryah, Zubair M. Ahmed

**Affiliations:** 1 Institute of Biotechnology and Genetic Engineering, The University of Sindh, Jamshoro 76090, Pakistan; priya_jarwar@hotmail.com; 2 Department of Molecular Biology & Genetics, Liaquat University of Medical and Health Sciences, Jamshoro 76090, Pakistan; compujin@gmail.com (S.A.S.); sriazuddin@som.umaryland.edu (S.R.); 3 Department of Otorhinolaryngology Head and Neck Surgery, School of Medicine, University of Maryland, Baltimore, MD 21201, USA; 4 Scientific Ophthalmic and Research Laboratory, Sindh Institute of Ophthalmology and Visual Sciences, Hyderabad 71500, Pakistan; yarmwaryah@hotmail.com; 5 Department of Pathology, Liaquat University of Medical and Health Sciences, Jamshoro 76090, Pakistan; ikramujjan@lumhs.edu.pk; 6 Department of Molecular Biology and Biochemistry, School of Medicine, University of Maryland, Baltimore, MD 21201, USA; 7 Department of Ophthalmology and Visual Sciences, School of Medicine, University of Maryland, Baltimore, MD 21201, USA

**Keywords:** cataract, *EPHA2*, hereditary congenital cataract, exome sequencing, inbred population, tyrosine kinase receptor, Eph receptor

## Abstract

Hereditary congenital cataract (HCC) is clinically and genetically heterogeneous. We investigated HCC that segregates in three inbred families (LUCC03, LUCC16, and LUCC24). Ophthalmological examinations revealed cataracts with variability related to the age of onset segregating in a recessive manner in these families. Exome sequencing of probands identified a novel homozygous c.2710delG;p.(Val904Cysfs*36) *EPHA2* variant in LUCC03 and a known homozygous c.2353G>A;p.(Ala785Thr) *EPHA2* variant in the other two recessive families. *EPHA2* encodes a transmembrane tyrosine kinase receptor, which is primarily involved in membrane-transport, cell-cell adhesion, and repulsion signaling processes. Computational structural modeling predicts that substitution of a threonine for an alanine p.(Ala785Thr) results in the formation of three new hydrogen bonds with the neighboring residues, which causes misfolding of EPHA2 in both scenarios. Insights from our study will facilitate counseling regarding the molecular and phenotypic landscape of *EPHA2*-related HCC.

## 1. Introduction

Hereditary congenital cataract (HCC) affects every 4 in 10,000 newborns in the United States, and accounts for 20% of blindness worldwide [1,2]. The phenotypic presentation of HCC is the opacification of the crystalline lens of the eyes. HCC can be subdivided according to the etiology, anatomical location within the lens (e.g., nuclear) and appearance (e.g., pulverulent) and are usually characterized by a combination of later two attributes [3]. HCC may present as an isolated trait or as part of a syndrome [4]. Non-syndromic CC follows various inheritance patterns: primarily autosomal dominant (accounts for up to 89% of the reported cases), X-linked inheritance that accounts for up to 10% of the cases, and autosomal recessive cataract is relatively rare with a prevalence of 7% in inbred families [5]. Pathological variants in crystalline encoding genes are the most frequent cause of HCC in Chinese populations [6], while the variants in connexin genes are more frequent in individuals with an Indian ethnicity [7]. However, as of August 2021, disease causing variants in 38 genes, including *EPHA2* (OMIM:176946), have been associated with the nonsyndromic HCC with a variable prevalence rate worldwide [8,9]. *EPHA2* encodes a transmembrane tyrosine kinase receptor belonging to the Eph receptor family, which is involved in membrane-transport and repulsion signaling processes [10]. EPHA2 receptor also participates in the cell adhesion, migration, and cell transformations from epithelial-to-mesenchymal cells—a fundamental process for the development, maintenance, and functioning of lens [11].

Both dominant and recessively inherited variants of *EPHA2* are known to cause cataract in endogamous families [12,13]. Variants of *EPHA2* are associated with different phenotypes and clinical presentations that manifest different morphologies including posterior polar opacities, nuclear opacities, cortical opacities, and total lens opacities [13]. As of August 2021, twenty-two disease causing variants of *EPHA2* have been reported [14]. Different types of variants of *EPHA2* have impact on the ligand-binding domain, epidermal growth factor-like domain, or tyrosine kinase catalytic activity of the encoded proteins, which ultimately leads to dysfunctional receptor and cataract phenotype [12,13].

As of August 2021, only 3 Pakistani families segregating *EPHA2* HCC-causing variants have been literature-documented [14]. In this present study, exome sequencing was used to identify the disease-causing variants in three large consanguineous Pakistani families with HCC. We identified two homozygous (c.2710delG, c.2353G>A) alleles of EPHA2 segregating with the HCC phenotype in these three families.

## 2. Results

**Figure 1:**
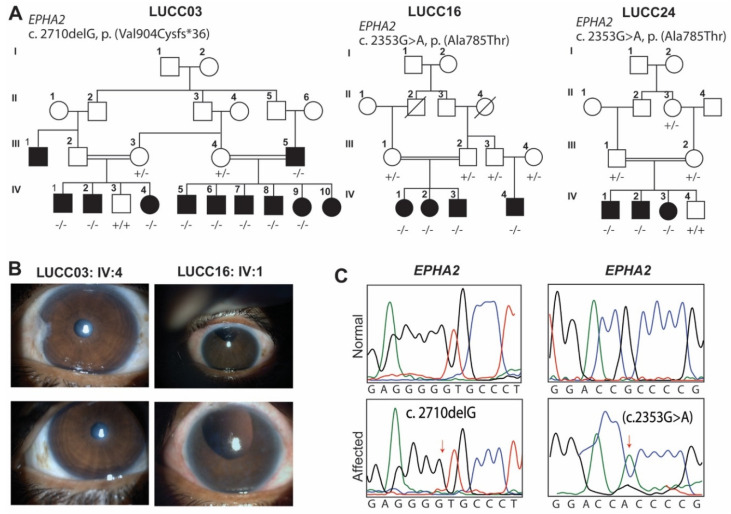
Cataract phenotype is associated with pathogenic variants of *EPHA2*. (**A**) Pedigrees of families LUCC03, LUCC16 and LUCC24. Double lines indicate consanguineous families, empty symbols represent unaffected individuals, and filled symbols affected individuals. The genotypes of the identified variants are also shown for each of the participating family members. All three families had recessive mode of inheritance. (**B**) Images of Slit lamp examination the LUCC03 IV:4 revealed nuclear cataract developed congenitally, while LUCC16 IV:1 had aphakic eyes. (**C**) Sanger sequencing DNA chromatograms of *EPHA2* variants found in this study.

As part of our ongoing efforts to ascertain and clinically and genetically characterize Pakistani families with HCC [15], three new large consanguineous families were enrolled from the remote areas of Sindh province of Pakistan (Figure 1A).

### 2.1. LUCC03 Family

**Figure 2:**
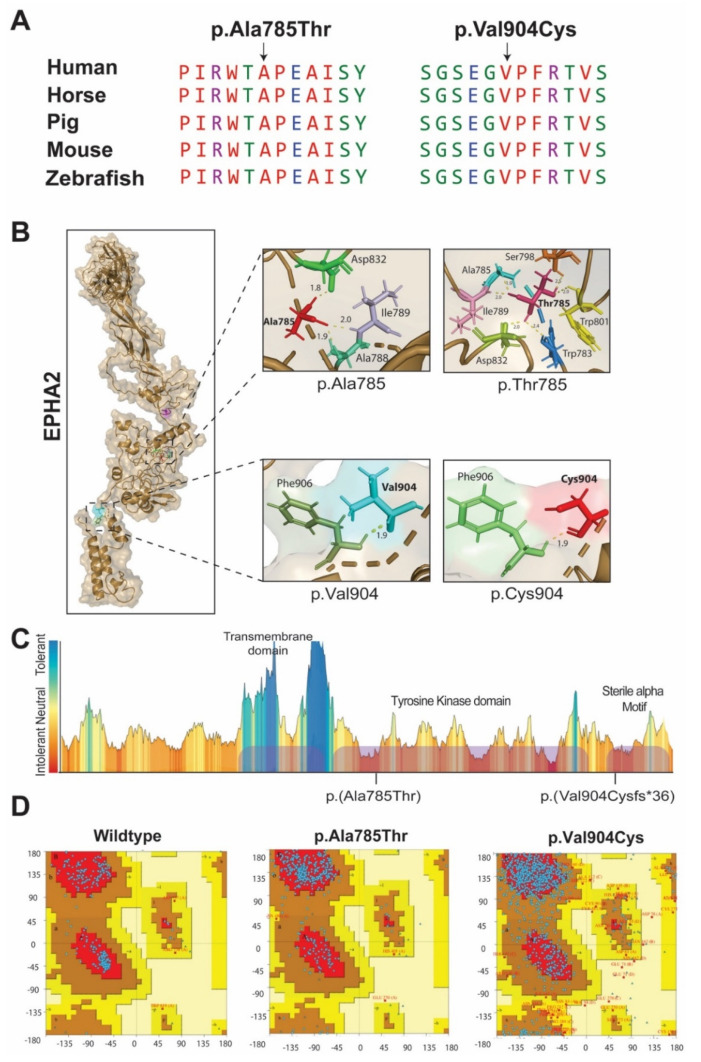
Protein modeling and bioinformatic analysis of identified variants of EPHA2. (**A**) Multiple sequence alignment of EPHA2 shows that all three residues are conserved across various vertebral species. (**B**) 3D Protein modeling of EPHA2 showing that wildtype and mutated residue of EPHA2 at position 904 do not depict any differences in bonding with neighboring residues, but mutated residue at position 785 is showing three extra hydrogen bonding with neighboring residues. (**C**) MetaDome health map shows that EPHA2 residue at position 785 and 904 are present in highly intolerant region. (**D**) Ramachandran plots for wildtype and mutated residues of EPHA2 revealed that as compared to 94.1% of the residue found in the allowed region for WT protein, 86.7% (EPHA2^p.Thr785^) and 67.6% (EPHA2^p.Cys904^) of the residues were present in allowed region for the EPHA2 harboring identified variant.

A four-generation pedigree consists of twenty-three members: including eleven individuals that have cataracts (Figure 1A,B). Family history revealed an autosomal recessive pattern of inheritance. Medical history questionaries indicated that the onset of cataracts was either at the time of birth or within first year of life (Supplementary Table S1). No other ocular-related abnormalities were noted. Exome sequencing of the proband DNA sample revealed a novel single base pair deletion c.2710delG in *EPHA2* (Figure 1C). Sanger sequencing of the identified variant confirmed the co-segregation of the variant with HCC in the family (Figure 1A). The c.2710delG variant was predicted to cause a shift in the codon reading frame [p.(Val904Cysfs*36)], leading to the loss of the evolutionary conserved carboxy tail (Figure 2A) and premature truncation of the encoded protein. The p.Val904 residue is part of the sterile alpha motif (SAM) of EPHA2 (Figure 2B,C), and it is predicted to truncate the motif. The Ramachandran plot, an in-silico tool used to assess the stereochemistry and geometry of the protein, further confirmed impact of p.(Val904Cysfs*96) on the secondary structure of EPHA2 (Figure 2D).

### 2.2. LUCC16 and LUCC24 Families

Both LUCC16 and LUCC24 had history of autosomal recessively inherited early-onset (Supplementary Table S1) cataract (Figure 1A,B). Although, these unrelated families were enrolled from different areas of Sindh a d belong to different ethnic subgroups, exome sequencing revealed a known c.2353G>A variant of *EPHA2* in both of their probands (Figure 1C). Sanger sequencing confirmed the segregation of the c.2353G>A variant with the cataract phenotype in both families (Figure 1A). The c.2353G>A variant was predicted to be damaging, had a low frequency in the gnomAD database (Table 1), and replaced an evolutionary conserved residue (p.(Ala785Thr)) of EPHA2 (Figure 2A). The replacement of the wild type alanine residue at position 785 with threonine, located within highly intolerant region of EPHA2 (Figure 2C), was predicted to induce formation of three extra hydrogen bonds with the neighboring residues (Figure 2B), which would likely impact the folding of encoded protein. However, the Ramachandran plot revealed comparable ranges of amino acids present in favorable regions between wild type and p.(Ala785Thr) variant-harboring mutant protein (Figure 2D).

## 3. Discussion

Hereditary congenital cataract is genetically heterogeneous. Variants in around 38 genes have been associated with nonsyndromic HCC; however, it is noteworthy that molecular causes of approximately 50% of the familial cases remain elusive. Goals of our o studies are to expand the genetic spectrum of HCC, improve the molecular diagnosis, aid in disease epidemiology, and quite possibly identify new targets for therapeutic interventions. Our current study expands the mutational spectrum of HCC in the Pakistani population. Through exome sequencing, we have identified two biallelic variants (c.2710delG; c.2353G>A) of *EPHA2* segregating with HCC in three inbred families.

Previously, a monoallelic two-base pair deletion (c.2915_2916delTG) in the penultimate exon of *EPHA2* had been documented in a Caucasian family with a posterior polar cataract [10]. The c.2915_2916delTG variant was predicted to cause reading frameshift and insert a cryptic C terminal peptide of 39 amino acid [10]. The mutant EPHA2 protein with the altered C tail had aberrant interaction with low molecular weight protein-tyrosin phosphatase: a negative regulator of EPHA2 signaling. Therefore, it likely acts as a ‘gain-of-function’ variant [10]. In contrast, the frameshifting variant we found in our study [c.2710delG; p.(Val904Cysfs*36)] was inherited recessively and the obligated carrier did not have cataract (Figure 1A). The p.(Val904Cysfs*36) variant is predicted to truncate the sterile alpha motif (SAM) of EPHA2 (Figure 2C)—a region essential for RNA binding activities besides other functions [16]. SAM domain might also be involved in the oligomerization, clustering of Eph-ephrin complex, and interactions with regulators [17]. Therefore, the p.(Val904Cysfs*36) variant that segregates in family LUCC03 is predicted to cause loss of SAM domain function, including RNA binding ability of EPHA2.

We also found a recurrent allele [c.2353G>A; p.(Ala785Thr)] of *EPHA2* in two unrelated families enrolled from the Sindh province of Pakistan. This variant was originally reported co-segregating with recessively inherited cataracts in a family enrolled from the Punjab province of Pakistan [12], indicating either a mutation hot spot or a founder allele. The p.(Ala785Thr) variant is located in the protein kinase domain of EPHA2, which is an essential region for protein-protein interaction and kinase activity. It is also predicted to impact the domain structure with consequences on the functionality of the protein [18]. Our findings might aid in further functional characterization of the EPHA2 in maintaining the transparent lens structure and improving the genetic basis of HCC in highly inbred Pakistani population.

## 4. Material Method

### 4.1. Ascertainment and Clinical Evaluation

After obtaining informed consent from all the participants, three consanguineous families were enrolled from different areas of Sindh, Pakistan. Detailed family history was recorded, and pedigrees were drawn to ascertain the mode of inheritance. All the affected individuals underwent detailed ophthalmologic examinations, and the clinical history of cataract was noted. The clinical findings were recorded by slit-lamp microscopy. Peripheral blood samples were collected for DNA extraction.

### 4.2. Sequencing and Bioinformatic Analysis

Exome sequencing was performed on the proband of each family by adopting the previously described method [19]. The NimbleGen EZ Exome V2 kit was used to synthesize the genomic libraries and was sequenced on an Illumina HiSeq4000. The data was filtered using the criteria previously described [20]. For the variants that passed our filtration strategy, Sanger sequencing was performed to evaluate the segregation with the phenotype in the participating families DNA samples.

Different bioinformatics tools were used to access the pathogenic effects of the variants and the potentially deleterious effects of the amino acid changes on the structure and the function of EPHA2. Clustal Omega multiple sequence alignment (https://www.ebi.ac.uk/Tools/msa/clustalo/, (accessed on 9 September 2021)) was used to evaluate the evolutionary conservation of the identified mutations. Varsome (https://varsome.com, (accessed on 9 September 2021)) was used for pathogenicity prediction of *EPHA2* variants, as in accordance with the American College of Medical Genetics and Genomics (ACMG) guidelines. Polyphen-2 (http://genetics.bwh.harvard.edu/pph2/, (accessed on 9 September 2021)), SIFT, and CADD scores (https://cadd.gs.washington.edu/score, (accessed on 9 September 2021)) were used to appraise the impact of the identified variants on the encoded proteins. 3D protein models were generated using the I-Tasser server (https://zhanglab.dcmb.med.umich.edu/I-TASSER/, (accessed on 9 September 2021)) and analyzed through PYMOL (http://pymol.org/2/, (accessed on 9 September 2021)). Their stereochemistry was assessed through PDBsum and Ramachandran plots. Finally, the protein tolerance map was plotted using MetaDome (https://stuart.radboudumc.nl/metadome/dashboard, (accessed on 9 September 2021)).

## Figures and Tables

**Table 1:** *EPHA2* variants, pathogenicity predictions and their ACMG classification.

cDNA Change	Protein Change	CADD	GnomAD	Mutation Taster	Polyphen2	SIFT	ACMG Classification (Criteria Used)	Reference
c.2353G>A	p.(Ala785Thr)	23.1	0.0003	Disease Causing	Probably Damaging	Damaging	Uncertain Significance (BP1, PP3, PP5)	[12]
c.2710delG	p.(Val904Cysfs*36)	N/A	0.000004	Disease Causing	N/A	N/A	Pathogenic (PVS1, PM2, PP3)	This study

N/A: Not available; CADD: Combined Annotation Dependent Depletion, https://cadd.gs.washington.edu/ (accessed on 9 September 2021). gnomAD: GnomAD browser, https://gnomad.broadinstitute.org (accessed on 9 September 2021). PVS1: Pathogenic very strong [null variant (nonsense, frameshift, canonical ±1 or 2 splice sites, initiation codon, single or multiexon deletion) in a gene where LOF is a known mechanism of disease)]. PM2: Pathogenic moderate 2 [Absent from controls (or at extremely low frequency if recessive) in Exome Sequencing Project, 1000 Genomes Project, or Exome Aggregation Consortium]. PP3: Pathogenic supporting 3 [Multiple lines of computational evidence support a deleterious effect on the gene or gene product (conservation, evolutionary, splicing impact, etc.)]. PP5: Pathogenic supporting 5 [Reputable source recently reports variant as pathogenic, but the evidence is not available to the laboratory to perform an independent evaluation]. BP1: Benign supporting 1 [Missense variant in a gene for which primarily truncating variants are known to cause disease].

## Data Availability

The variants reported in this study have been deposited in the ClinVar database.

## Supplementary Materials

The following are available online at https://www.mdpi.com/article/10.3390/ijms221910655/s1.
