# Supplementary material for: Biallelic Variants in EPHA2 Identified in Three Large Inbred Families with Early-Onset Cataract
Source: Int J Mol Sci. 2021 Sep 30;22(19):10655. doi: 10.3390/ijms221910655 (PMC8508826; doi:10.3390/ijms221910655)
Supplement: Supplementary file 1 [file ijms-22-10655-s001.zip › ijms-1399884-supplementary.pdf]

**Supplementary Table S1: Medical history of congenital cataract in families segregating *EPHA2* alleles**

| Family & *EPHA2* variant | Sex | Onset Age | Age of Diagnosis | Age at surgery | |
|---|---|---|---|---|---|
| | | | | **Right eye** | **Left eye** |
| **LUCC03:** c.2710delG; p.(Val904Cysfs*36) | | | | | |
| III:5 | M | Congenital | 10 year | 12 year | Not operated |
| IV:1 | M | Congenital | 5 year | 9 year | Not operated |
| IV:2 | M | Congenital | 8 year | Not operated | |
| IV:4 | F | Congenital | 5 year | Not operated | |
| IV:5 | M | Congenital | 3 year | 17 year | 17 year |
| IV:6 | M | Congenital | 2 year | 8 year | Not operated |
| IV:7 | M | Congenital | 5 year | 9 year | 9 year |
| IV:8 | M | Congenital | 5 year | 8 year | Not operated |
| IV:9 | F | Congenital | 4 year | 5 year | Not operated |
| IV:10 | F | Congenital | 3 year | 6 year | 6 year |
| **LUCC16:** c.2353G>A; p.(Ala785Thr) | | | | | |
| IV:1 | F | Congenital | 3 year | 6 year | 6 year |
| IV:2 | F | Congenital | 2 year | 3 year | Not operated |
| IV:3 | M | Congenital | 6 month | 1 year | 1.5 year |
| IV:4 | M | Congenital | 1 year | 1.5 year | 2 year |
| **LUCC24:** c.2353G>A; p. (Ala785Thr) | | | | | |
| IV:1 | M | Congenital | 3 month | 5 year | 4 year |
| IV:2 | M | Congenital | 3 month | 3 year | 3.5 Year |
| IV:3 | F | Congenital | 3 month | Not operated | |
